# Indian Hedgehog regulates senescence in bone marrow-derived mesenchymal stem cell through modulation of ROS/mTOR/4EBP1, p70S6K1/2 pathway

**DOI:** 10.18632/aging.102958

**Published:** 2020-04-01

**Authors:** Mahmoud Al-Azab, Bing Wang, Abdalkhalig Elkhider, Williams Walana, Weiping Li, Bo Yuan, Yunshan Ye, Yawei Tang, Marwan Almoiliqy, Salah Adlat, Jing Wei, Yan Zhang, Xia Li

**Affiliations:** 1 Department of Immunology, College of Basic Medical Science, Dalian Medical University, Liaoning, China; 2 Department of Immunology, Guangzhou Institute of Pediatrics, Guangzhou Women and Children’s Medical Center, Guangzhou Medical University, Guangzhou, China; 3 Department of Rheumatology and Immunology, The Second Affiliated Hospital of Dalian Medical University, Liaoning, China; 4 Department of Pharmacology, College of Pharmacy, Dalian Medical University, Liaoning, China; 5 Key Laboratory of Molecular Epigenetics of MOE, School of Life Science, Northeast Normal University, Changchun, Jilin Province, China; 6 Department of Clinical Microbiology, University for Development Studies, Tamale, Ghana

**Keywords:** Indian hedgehog, aging, differentiation, mesenchymal stem cell, mammalian target of rapamycin

## Abstract

Premature senescence of bone marrow-derived mesenchymal stem cells (BMSC) remains a major concern for their application clinically. Hedgehog signaling has been reported to regulate aging-associated markers and MSC skewed differentiation. Indian Hedgehog (IHH) is a ligand of Hedgehog intracellular pathway considered as an inducer in chondrogenesis of human BMSC. However, the role of IHH in the aging of BMSC is still unclear. This study explored the role IHH in the senescence of BMSC obtained from human samples and senescent mice. Isolated BMSC were transfected with IHH siRNA or incubated with exogenous IHH protein and the mechanisms of aging and differentiation investigated. Moreover, the interactions between IHH, and mammalian target of rapamycin (mTOR) and reactive oxygen species (ROS) were evaluated using the corresponding inhibitors and antioxidants. BMSC transfected with IHH siRNA showed characteristics of senescence-associated features including increased senescence-associated β-galactosidase activity (SA-β-gal), induction of cell cycle inhibitors (p53/p16), development of senescence-associated secretory phenotype (SASP), activation of ROS and mTOR pathways as well as the promotion of skewed differentiation. Interestingly, BMSC treatment with IHH protein reversed the senescence markers and corrected biased differentiation. Moreover, IHH shortage-induced senescence signs were compromised after mTOR and ROS inhibition. Our findings presented anti-aging activity for IHH in BMSC through down-regulation of ROS/mTOR pathways. This discovery might contribute to increasing the therapeutic, immunomodulatory and regenerative potency of BMSC and introduce a novel remedy in the management of aging-related diseases.

## INTRODUCTION

Eukaryotic cell aging is an irreversible loss of growth and proliferation which is maintained by intrinsic and extrinsic factors after unceasing cellular replication or surrounding stress. Unbalance in genes expressions of growth regulatory proteins such as P53, P16, and P21, morphological changes, cell cycle arrest, and senescence-associated β-galactosidase (SA β-gal) activities are the main characters in cellular senescence [1]. Usage of mesenchymal stem cells (MSC), especially bone marrow-derived MSC (BMSC), in the treatment of rheumatic diseases, and regenerative medicine has acquired both treatment and curative potential. However, MSC’s early aging *in vitro* remains a challenge which seems to restrain the efforts of scientists and physicians in MSC research and clinical applications. It has been reported that the aging of BMSC interrupts its therapeutic activities such as anti-inflammatory cytokines production reduction and decrease in ability to repair bone fractures [2–5]. One of the *In vivo* signs of cell aging is skewed differentiation of MSC to adipocyte at the cost of osteoblast, which is considered a core effector in osteoporosis [6]. Thus, keeping BMSC young, preventing aging, and maintaining physiologically balanced differentiation during *in vitro* or *in vivo* proliferation are important fundamental requirements in the clinical applications of BMSC and management of aging-related diseases.

Hedgehog signaling is an intracellular pathway with three protein ligands; sonic Hedgehog (Shh), Indian Hedgehog (IHH), and desert Hedgehog (Dhh). Patched1/2 (PTCH1/2) and smoothened (Smo) are trans-membrane receptors which mediate the action of hedgehog members on their target, glioblastoma gene [7]. It has been reported that Hedgehog signaling orchestrates MSC differentiation and prevents the prominent marker of aging, skewed differentiation, by inducing osteogenesis at the cost of adipogenesis [8]. Meanwhile, a core player in aging, P53 pathway, may have an effective interaction with Hedgehog signaling [9]. In addition, IHH, a Hedgehog homolog related to cartilage and bone formation is considered as an inducer in chondrogenesis of human MSC [10]. Moreover, it is reported that the IHH gene transfection could inhibit cartilage senescence [11]. On the other hand, there are a variety of factors that stimulate a cell to be in senescence mood, and these include stimulation by inflammatory cytokines, poor cell-to-cell contact, nutrient deficiency, and disturbance in the regulation of intracellular signaling pathways such as STAT3, NF-kB, Akt, and PI3K [12–15]. Thus, exploring the interaction of IHH with the above-mentioned parameters may contribute to the understanding of the senescence machinery.

Oxidative stress is one of the major inducers of aging in BMSC, where reactive oxygen species (ROS) have been reported as a promotor of adipogenic differentiation and a repressor of osteogenic differentiation in MSC [8]. Additionally, ROS decrease the ability of BMSC to maintain the hematopoiesis compartment in aged mice [16]. Indeed, it has been recommended that optimizing intracellular and mitochondrial ROS of MSC is required to obtain an optimum effect in treatment by MSC [17]. Therefore, exploration of pathways that regulate the releasing of ROS may provide clues to improve immunoregulatory properties of BMSC in tissue engineering, regenerative medicine, and treatment of autoimmune diseases.

The serine/threonine protein kinase of phosphatidylinositol-3-OH kinase (PI3K) family, mammalian target of rapamycin (mTOR) has two functional proteins, mTOR complex 1 (TORC1) and mTOR complex 2 (TORC2). The mTOR pathway is a growth and metabolism regulator through its three downstream effectors, eukaryotic translation initiation factor 4E-binding protein 1 (4EBP1), phosphorylated ribosomal S6 kinase 1/2 (p70S6K1/2) and Akt [18, 19]. Although it is reported that mTOR signaling plays a role in the skewed differentiation of MSC and aging [20], the effect of its inhibition in MSC differentiation is still a subject of conflict. Moreover, the inhibition of this pathway clinically may lead to the desired output in the treatment of aging-related diseases, especially osteoporosis and arthritis [21]. However, the role of IHH and mTOR interaction in the aging of BMSC is still an open issue.

In this study, we observed that IHH has regulatory activities in BMSC senescence and differentiation via controlling the mTOR downstream substrates, 4EBP1 and p70S6K1/2. Herein, we showed that IHH down-regulates oxidative stress in order to protect BMSC from senescence by maintaining the desired differentiation.

## RESULTS

### IHH is decreased at both transcription and protein levels in senescent BMSC

**Figure 1:**
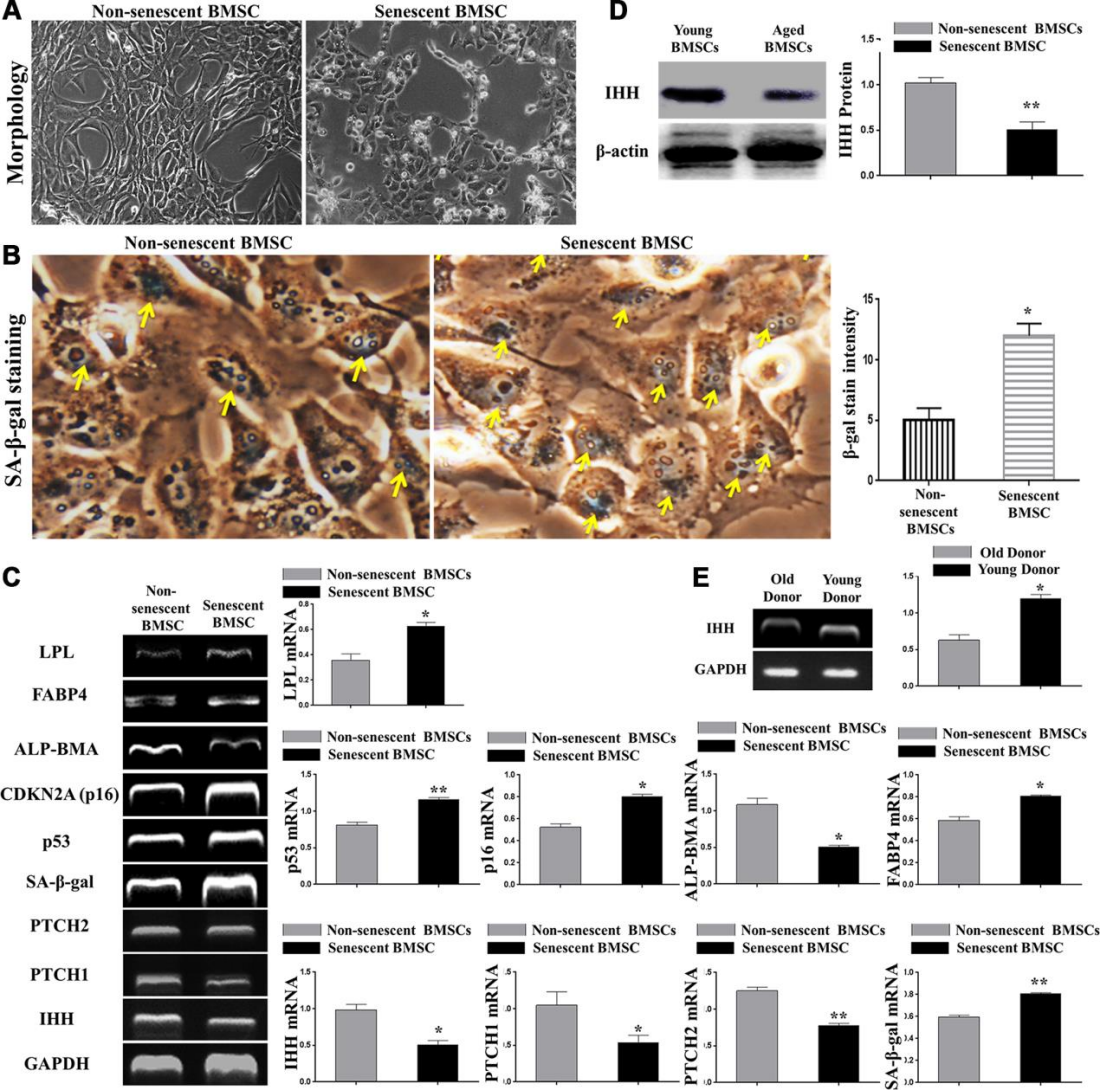
**IHH signaling pathway downregulated in aged BMSC**. (**A**) Morphology of non-senescent and senescent BMSC. (**B**) Non-senescent and senescent BMSC stained by SA-β-gal stain. (**C**) BMSC (n = 4) were incubated with or without senescence-induction medium for 8 days. IHH, PTCH1/2, SA-β-gal, p53, p16, ALP-BMA, FABP4, and LPL genes expressions were measured by RT-PCR. GAPDH was used as a housekeeping gene. (**D**) Isolated BMSC (n = 4) were incubated with or without senescence-induction medium for 8 days. IHH protein expression was measured by Western Blot. β-actin was used as an internal control. (**E**) Isolated BMSC from young and old donors (n = 4) were analyzed for IHH gene expression by RT-PCR. GAPDH was used as a housekeeping gene. Results presented as mean ± SEM. *P <0.05, **P < 0.01.

H_2_O_2_-induced senescence was displayed by severe morphological changes (Figure 1A), increased SA-β-gal-stained BMSC count (Figure 1B), increased expression of aging-related genes, p53, p16, SA-β-gal, decreased ALP-BMA activity, and increased adipogenic markers, FABP4 and LPL (Figure 1C). Because of the importance of IHH in embryonic growth and differentiation, we proposed its involvement and impact in aging. We, therefore, searched the main members of the IHH signaling pathway in non-senescent and senescent BMSC. We amplified the gene expression of cells using RT-PCR and the results revealed a significant decrease in IHH gene expression in senescent BMSC compared to non-senescent cells (Figure 1C). Due to their core role in the Hedgehog pathway, we determined PTCH1/2 receptors expressions. Consistent with the previous results, both receptors were down-regulated in senescent BMSC (Figure 1C). In accordance with PCR results, western blot results for IHH protein (Figure 1D) showed that senescent BMSC was characterized by decreased IHH protein synthesis. In addition, we observed a decreased IHH gene expression in BMSC isolated from aged donors compared to young donors (Figure 1E). These results suggested that BMSC lost their ability to synthesize IHH as they aged, and therefore IHH could have an essential task in the regulation of BMSC senescence and differentiation.

### Silencing of IHH promoted aging and altered paracrine-related genes in BMSC

**Figure 2:**
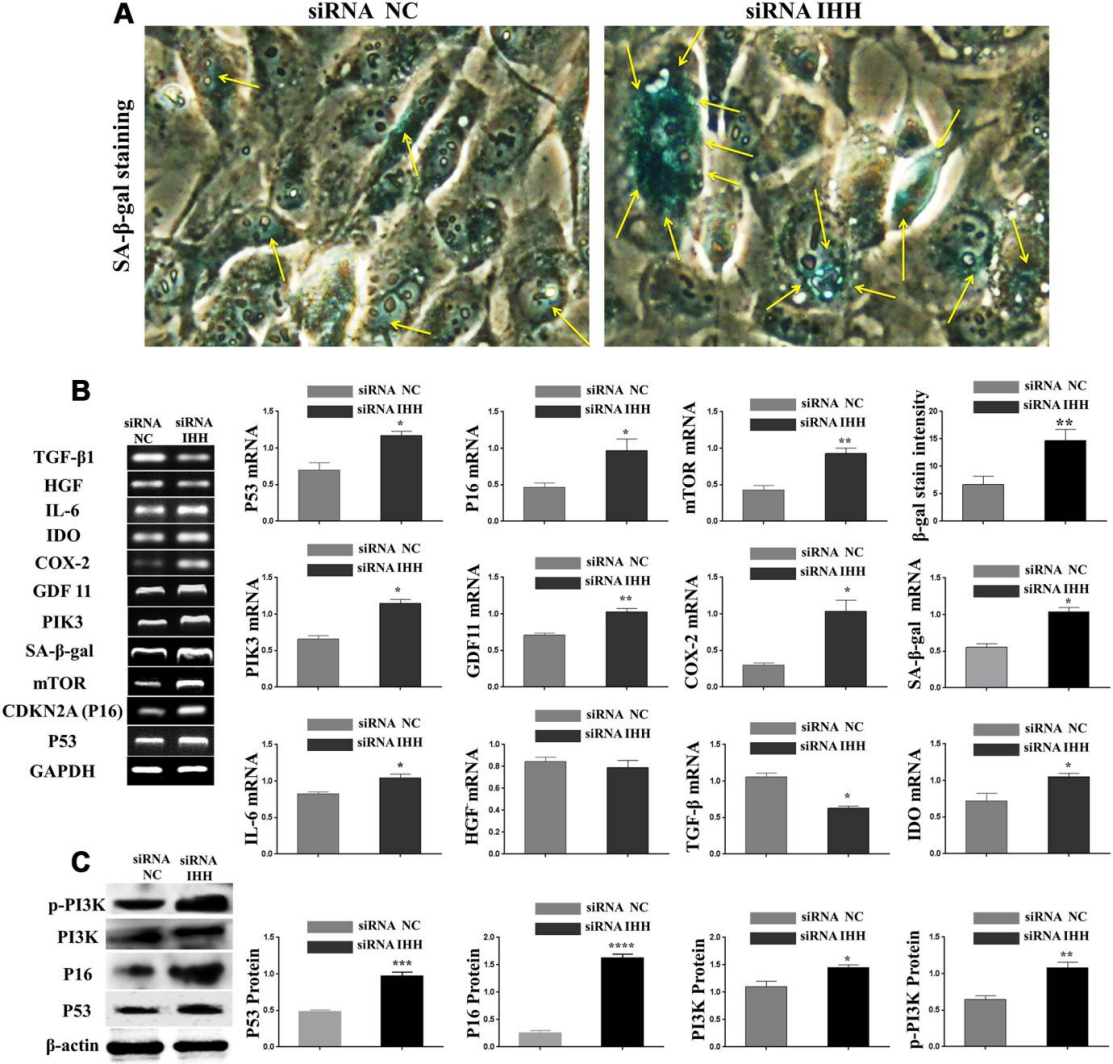
**IHH knockdown induced BMSC senescence.** (**A**) BMSC transfected with NC siRNA and IHH siRNA stained by SA-β-gal stain. (**B**) BMSC (n = 5) were transfected with siRNA negative control or siRNA IHH for 24hours. TP53, CDKN2A, SA-β-gal, mTOR, PIK3, GDF11, COX-2, IDO1, IL-6, HGF, and TGFβ genes expressions were measured by RT-PCR. GAPDH was used as a housekeeping gene. (**C**) BMSC (n = 5) were transfected with siRNA negative control or siRNA IHH for 48hours. P53, P16, PI3K, and p-PI3K proteins expressions were measured by Western Blot. β-actin was used as an internal control. Results presented as mean ± SEM. *P <0.05, **P < 0.01, ***P < 0.001, ****P.

To assess the potential role of IHH in the aging of BMSC, IHH expression was depleted by siRNA silencing. An obvious IHH knockdown was presented by RT-PCR and western blot (Supplementary Figure 2A, 2B). Because existing literature supported the involvement of P53, P16, mTOR, SA-β-gal, PI3K and GDF11 genes in aging, we investigated their expressions in the different states of BMSC. Surprisingly, the count of BMSC cells stained by SA-β-gal stain increased in cells treated with IHH siRNA (Figure 2A). Similarly, the results of RT-PCR revealed induced expressions for all the above-mentioned genes in BMSC transfected with IHH siRNA compared to the negative control (Figure 2B). In a related issue, the role of paracrine secretions in immunomodulatory and regenerative capacity is a major pathway in the therapeutic usage MSC. It was therefore deemed necessary to evaluate the immunomodulatory potency of BMSC expressing reduced IHH, and to investigate their relationship with the aging process. RT-PCR results showed that paracrine-related genes COX-2, IDO, and IL-6 were significantly induced, while TGF-β was significantly down-regulated, and HGF experience moderate down-regulation in IHH siRNA transfected BMSC (Figure 2B). For further confirmation, we selected the main markers, P53, P16, and PI3K for western blot examination. Interestingly, the results obtained supported the SA-β-gal stain and RT-PCR results as the three proteins were up-regulated in IHH siRNA transfected BMSC (Figure 2C). Morphologically, the IHH siRNA transfected BMSC showed more transparency, slight enlargement, and decreased in cell count compared to the negative control (Supplementary Figure 2D). Our results demonstrated that IHH might have an anti-aging effect, and its inhibition altered paracrine secretions of BMSC.

### IHH knockdown inhibited proliferation and activated cell cycle arrest and aging-related signaling pathways in BMSC

**Figure 3:**
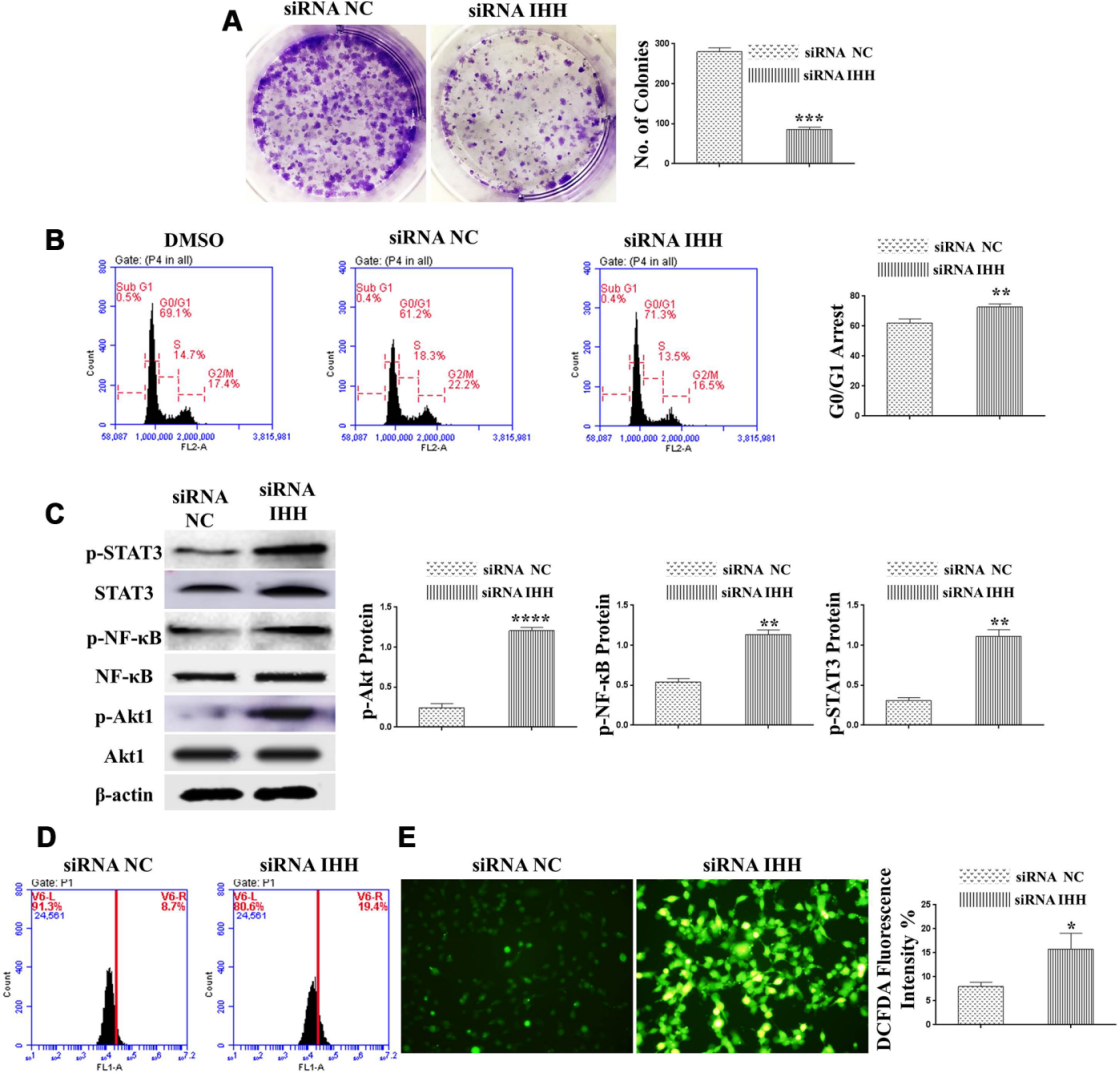
**Silencing of IHH induced CFU inhibition, cell cycle arrest, and aging-related signaling pathways in BMSC.** (**A**) BMSC (n = 5) were transfected for 24hours and then incubated in 10% FBS in MEM-ALPHA medium for 12 days. Colonies were visualized after staining by 0.02% crystal violet stain. (**B**) BMSC (n = 5) were transfected with siRNA negative control, siRNA IHH or DMSO for 24hours. Fixed cells stained by PI and RNase A, and then analyzed by flow cytometry for cell cycle distribution (**C**) BMSC (n = 5) were transfected with siRNA negative control or siRNA IHH for 48hours. Akt1, p-Akt1, NF-κB, p-NF-κB, STAT3, and p-STAT3 proteins expressions were measured by Western Blot. β-actin was used as an internal control. (**D**) BMSC (n = 5) were transfected with siRNA negative control or siRNA IHH for 24hours then stained by DCFHDA (5 μM). The fluorescent intensity of ROS was measured by flow cytometry and immunofluorescence microscopy (**E**). Results are shown as mean ± SEM. *P <0.05, **P < 0.01, ***P < 0.001, ****P < 0.0001.

For further assessment of the role of IHH in BMSC aging, we analyzed IHH siRNA transfected BMSC for proliferation capacity and markers of cellular pathways relevant to aging mechanisms. Fibroblast colony forming assay results revealed that IHH siRNA-transfected BMSC failed to form colonies contrary to the negative control (Figure 3A). Consistent with colony forming assay results, propidium iodide DNA staining was employed for cell cycle assay by FACS, and G0/G1 cell cycle arrest was associated with BMSC of IHH siRNA (Figure 3B). Molecularly, immunoblotting assay results showed phosphorylation up-regulation of AKT1, NF-κB, and STAT3 was associated with IHH shortage (Figure 3C). Since oxidative stress was well recognized as a promoter of aging and was involved in the IHH signaling pathway, we assessed ROS generation using flow cytometry. Increased ROS generation in the transfected BMSC was observed compared to the negative control (Figure 3D). Alternatively, ROS generation results were consistent by using immunofluorescence technique (Figure 3E). Collectively, our findings showed that IHH could protect BMSC from aging by promoting proliferation and modulating Akt1, NF-κB, and STAT3 signaling pathways, as well as down-regulating oxidative stress

### BMSC distorted differentiation was associated with IHH knockdown

**Figure 4:**
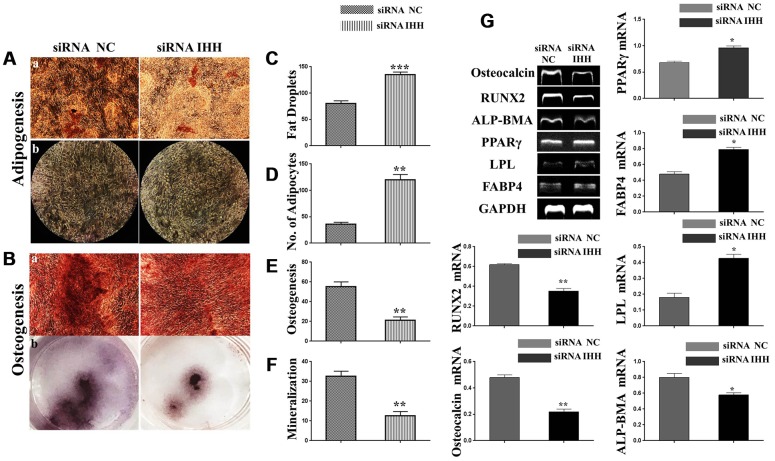
**Skewed differentiation was induced in IHH-depleted BMSC.** (**Aa**, **b**), (**C** and **D**) BMSC (n = 5) were transfected with siRNA negative control or siRNA IHH for 24hours then incubated in adipogenesis differentiation medium for 21 days. Adipocytes were visualized under inverted light microscope and adipogenesis measured by staining fat droplets using Oil-Red-O stain. (**Ba**, **b**), (**E**, **F**) BMSC (n = 5) were transfected with siRNA negative control or siRNA IHH for 24hours then incubated in osteogenic differentiation medium for 21 days. Osteogenesis and Ca_2_ mineralization measured using Alizarine-Red-S stain. (**G**) RT-PCR for adipogenesis markers, PPARγ, LPL, and FABP4, and osteogenesis markers, ALP-BMA, RUNX2, and osteocalcin in BMSC with and without IHH siRNA. GAPDH was used as a housekeeping gene. Results have shown as mean ± SEM. *P <0.05, **P < 0.01, ***p <0.001.

Normal differentiation of BMSC is a major advantage that endows the regenerative potency in stem cells. Adipogenic and osteogenic differentiations are balanced mechanisms within physiologically permissible limits, where there is the required increase in osteogenesis than adipogenesis, it is seen as an indicator of functionally normal BMSC. On the contrary, the converse of this mechanism is a sign of aging. To assay for adipogenesis, we incubated the two kinds of BMSC in an adipogenic differentiation medium for 21 days. After that, we applied Oil-Red-O staining protocol which revealed increased red-yellow fat droplets in IHH siRNA-transfected BMSC (Figure 4Aa, 4C). Additionally, we observed a significant increase in adipocytes within the IHH siRNA-transfected BMSC compared to the non-transfected counterpart (Figure 4Ab, 4D). For osteogenesis assessment, the IHH siRNA-transfected BMSC demonstrated decreased red-orange calcium deposition (Figure 4Ba, 4E) and mineralization (Figure 4Bb, 4F). Consistently, RT-PCR results showed increased genes expression of adipogenesis markers, PPARγ, LPL, and FABP4, and decreased genes expression of osteogenesis markers, ALP-BMA, RUNX2, and osteocalcin in IHH siRNA-transfected BMSC (Figure 4G). These outcomes suggested IHH promoted osteogenesis at the cost of adipogenesis, thereby protecting BMSC from aging.

### IHH suppressed senescence of BMSC

**Figure 5:**
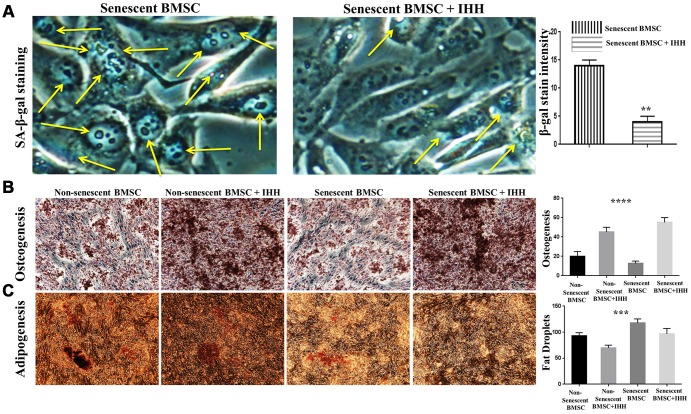
**IHH alleviated senescence and induced proper differentiation in BMSC.** (**A**) Senescent BMSC (n=5) incubated with and without IHH for 24 hrs, then stained by SA-β-gal stain. (**B**) Non-senescent and senescent BMSC (n = 5) were incubated in osteogenic differentiation medium with and without IHH for 21 days. Osteogenesis measured using Alizarine-Red-S stain. (**C**) Non-senescent and senescent BMSC (n = 5) were incubated in adipogenesis differentiation medium with and without IHH for 21 days. Adipogenesis measured by staining fat droplets using Oil-Red-O stain. All data obtained are indicated as mean ± SEM. **P < 0.01, ***p <0.001, ****p <0.0001.

**Figure 6:**
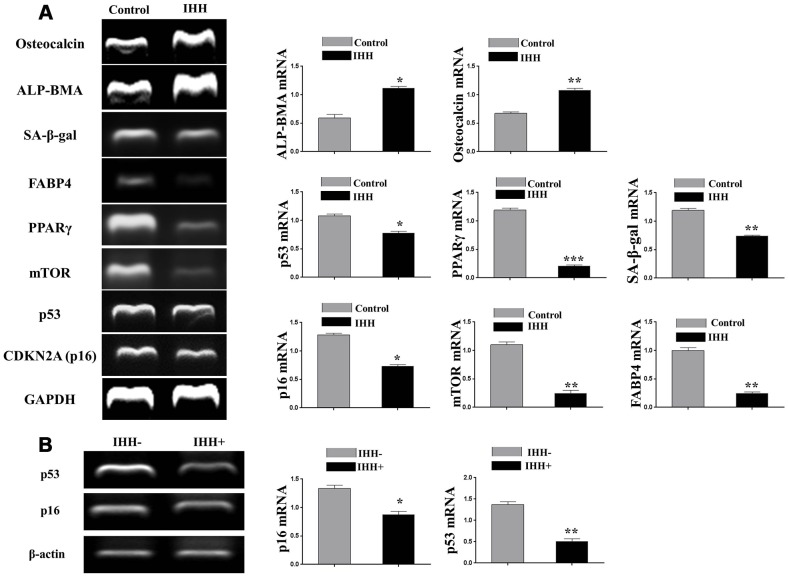
**IHH reversed aging-related genes and promoted genes of proper differentiation.** (**A**) BMSC (n = 5) were incubated with and without IHH for 24hours. Aging-related genes, TP53, CDKN2A, SA-β-gal, and mTOR, adipogenesis markers, PPARγ and FABP4, and osteogenesis markers, ALP-BMA, and osteocalcin genes expressions were measured by RT-PCR. GAPDH was used as a housekeeping gene. (**B**) BMSC from SAMP8 mice (n = 4) were incubated with and without IHH for 24hours. Aging-related genes, p16 and TP53 were measured by RT-PCR. β-actin was used as a housekeeping gene. All data obtained are indicated as mean ± SEM. *P <0.05, **P < 0.01, ***p <0.001.

In order to confirm the above results, we decided to study the different aspects of BMSC senescence and differentiation in the presence of rIHH protein. Thus, we incubated BMSC with rIHH and then analyzed the main markers of senescence and differentiation. Interestingly, treatment of senescent BMSC with rIHH decreased the count of SA-β-gal stained cells (Figure 5A) and adipogenesis (Figure 5C), and induced osteogenesis (Figure 5B). In addition, non-senescent BMSC treated with rIHH experienced increased osteogenesis (Figure 5B) and decreased adipogenesis (Figure 5C). Consistently, aging-related genes, p16, p53, SA-β-gal, and mTOR were down-regulated after treatment with rIHH (Figure 6). Moreover, RT-PCR results presented increased osteogenesis markers, BMA-ALP, and osteocalcin, and decreased adipogenesis markers; PPARγ and FABP4 in rIHH-treated BMSC (Figure 6A). Furthermore, CD73, CD90, and CD140a of BMSC were analyzed after rIHH treatment, however we realized that rIHH couldn’t affect the BMSCs CD markers expressions (Supplementary Figure 3). More important, treatment of BMSC isolated from Senescence Accelerated Mouse Prone-8 (SAMP8) mouse line by rIHH decreased aging-related genes, p16 and p53 (Figure 6B). These findings suggested that exogenous IHH reduces senescence and corrected skewed differentiation in BMSC.

### IHH depletion-induced BMSC senescence was suppressed by inhibition of mTOR and ROS

**Figure 7:**
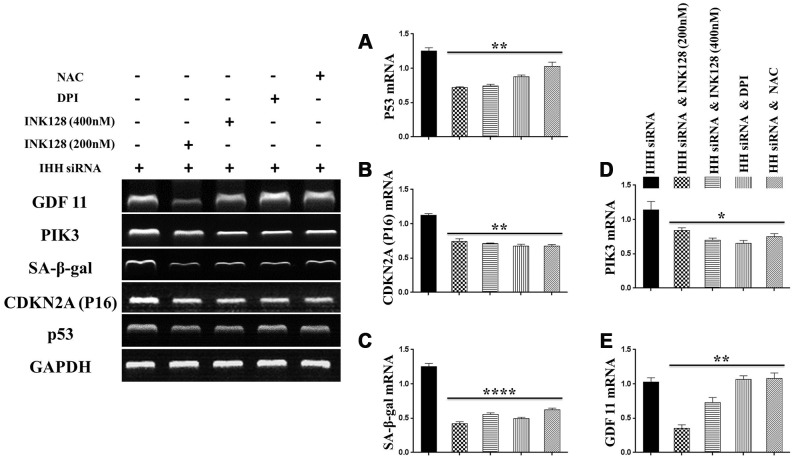
**IHH shortage-induced aging-associated genes suppressed by mTOR and ROS inhibition in BMSC.** siRNA IHH-transfected BMSC (n=5) were incubated with or without INK128, DPI, or NAC for 24hours. TP53 (**A**), CDKN2A (**B**), SA-β-gal (**C**), PIK3 (**D**) and GDF11 (**E**) genes expressions were measured by RT-PCR. GAPDH was used as a housekeeping gene. All results were normally distributed and shown as mean ± SEM. *P <0.05, **P < 0.01, ***p <0.001, ****p <0.001.

**Figure 8:**
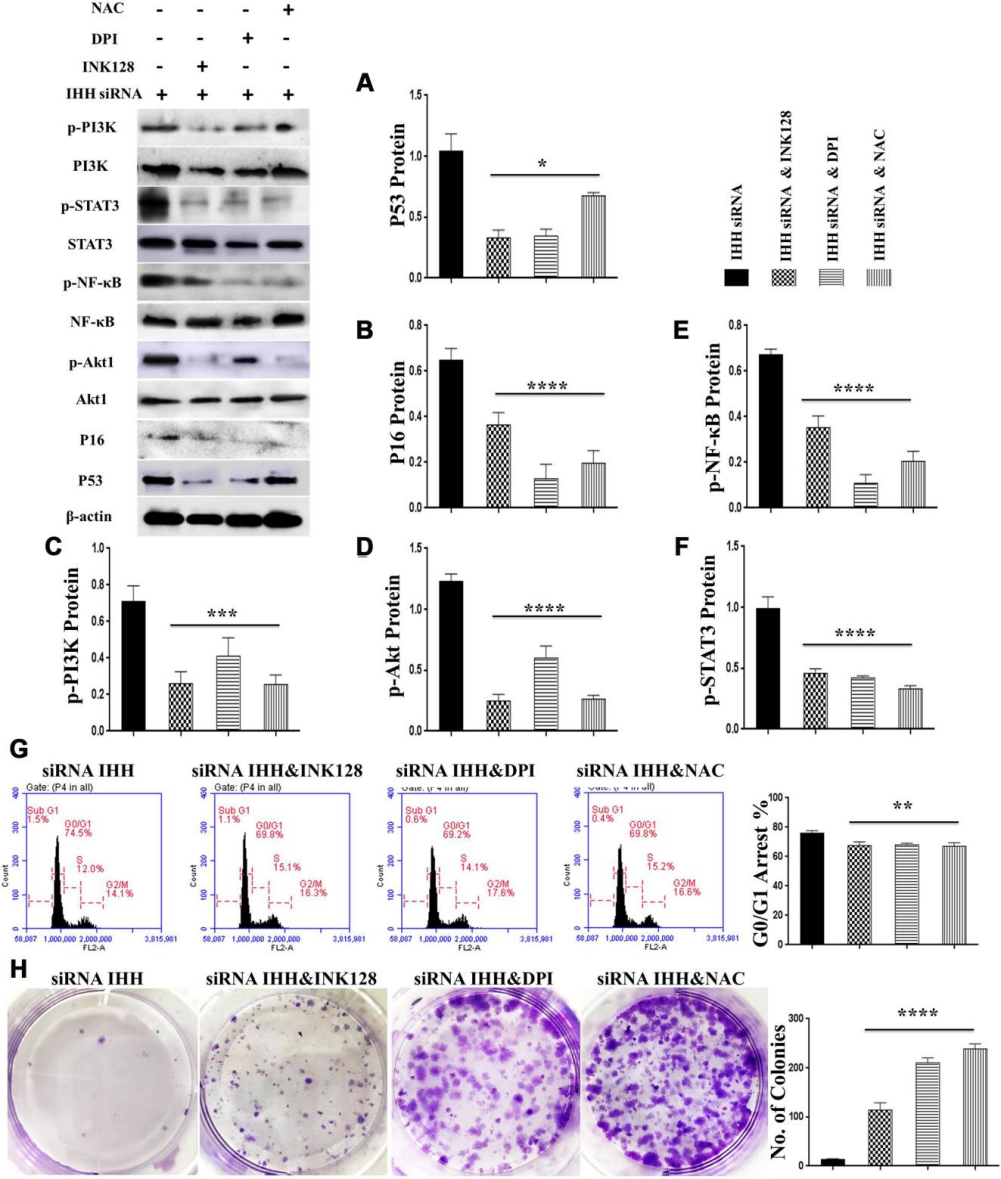
**mTOR and ROS inhibition reversed IHH depletion-induced senescence-related signaling pathways, cell cycle arrest, and inhibited CFU.** siRNA IHH-transfected BMSC (n=5) were incubated with or without INK128, DPI, or NAC for 48hours. P53 (**A**), P16 (**B**), PI3K, p-PI3K (**C**), Akt1, p-Akt1 (**D**), NF-κB, p-NF-κB (**E**), STAT3, and p-STAT3 (**F**) proteins expressions were measured by Western Blot. β-actin was used as an internal control. (**G**) BMSC (n = 5) were treated with or without INK128, DPI, or NAC in presence of siRNA IHH for 24hours. Fixed cells stained by PI and RNase A, and then analyzed by flow cytometry for cell cycle distribution. (**H**) BMSC (n = 5) were treated with or without INK128, DPI, or NAC in presence of siRNA IHH for 24hours and then incubated in 10% FBS in MEM-ALPHA medium for 12 days. Colonies were visualized after staining with 0.02% crystal violet stain. All results were normally distributed and shown as mean ± SEM. *P <0.05, **p <0.01, ***p <0.001, ****p <0.001.

**Figure 9:**
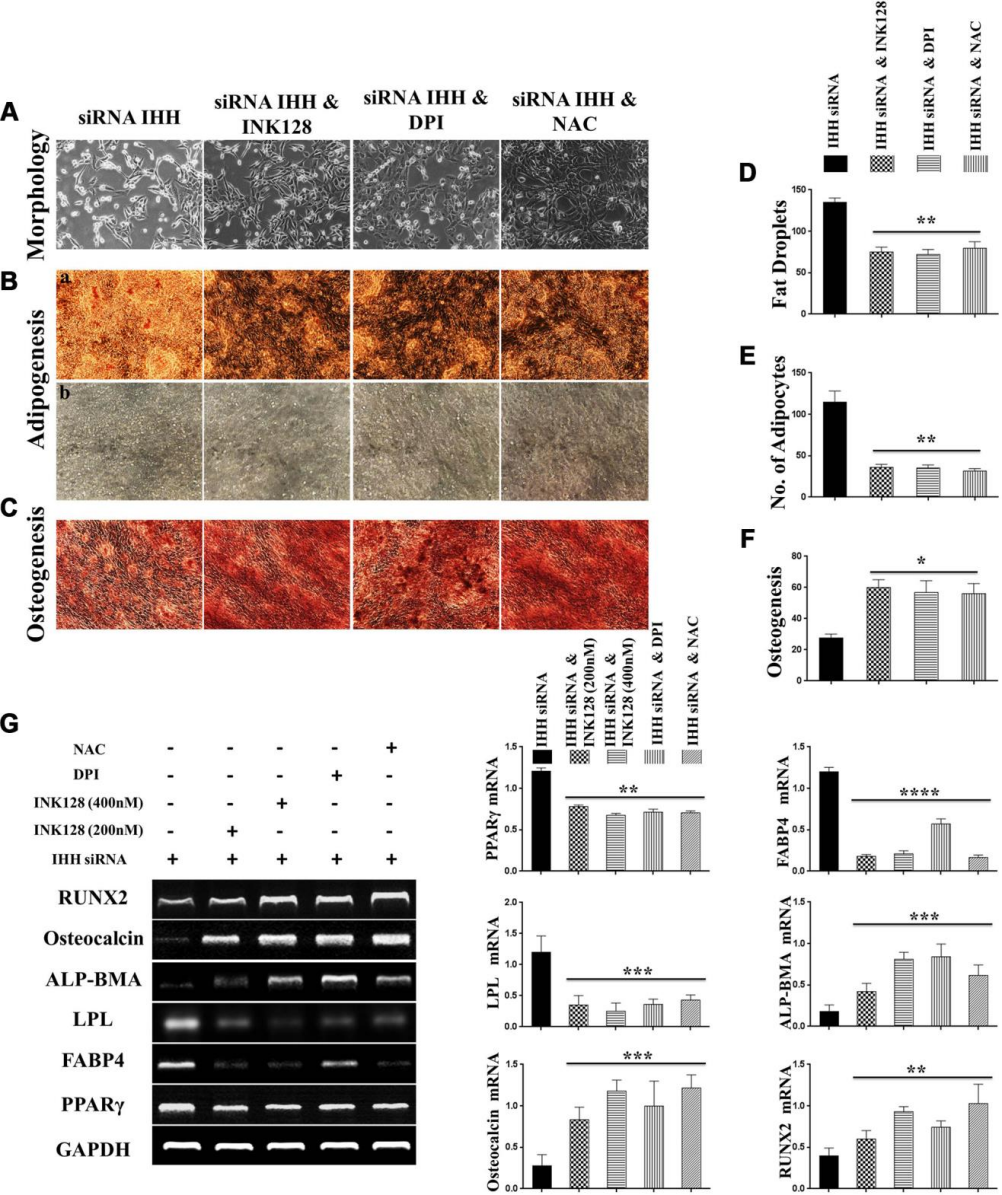
**Biased differentiation induced by IHH shortage was corrected after mTOR and ROS inhibition in BMSC.** (**A**) Morphology of siRNA IHH BMSC with and without INK128, DPI, or NAC. (**Ba**, **b**), (**D**, **E**) siRNA IHH-transfected BMSC (n=5) were incubated with or without INK128, DPI, or NAC for 24hours and then incubated in adipogenesis differentiation medium for 21 days. Adipocytes were visualized under inverted light microscope and adipogenesis measured by staining fat droplets using Oil-Red-O stain. (**C** and **F**) siRNA IHH-transfected BMSC (n=5) were incubated with or without INK128, DPI, or NAC for 24hours and then incubated in osteogenic differentiation medium for 21 days. Osteogenesis measured using Alizarine-Red-S stain. (**G**) RT-PCR for adipogenesis markers, PPARγ, LPL, and FABP4, and osteogenesis markers, ALP-BMA, RUNX2, and osteocalcin in IHH-depleted BMSC after incubation with or without INK128, DPI, or NAC for 24hours. GAPDH was used as a housekeeping gene. All results presented as mean ± SEM. *P <0.05, **P < 0.01, ***p <0.001, ****p <0.0001.

**Figure 10:**
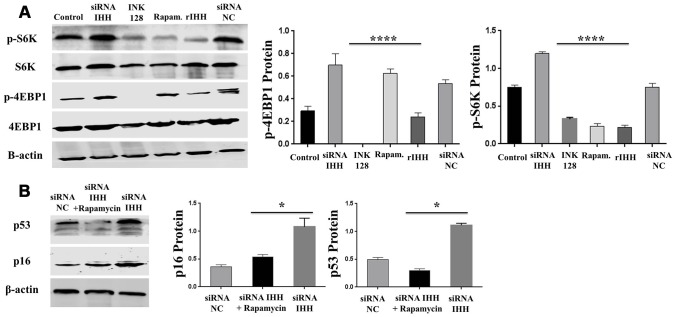
**IHH inhibited phosphorylation of 4EBP1 and p70S6K1/2 in BMSC.** (**A**) BMSC (n = 5) were transfected with siRNA negative control, siRNA IHH, rIHH, INK128, or rapamycin for 48hours. 4EBP1 and p70S6K1/2 total and phosphorylated proteins expressions were measured by Western Blot. β-actin was used as an internal control. (**B**) siRNA IHH-transfected BMSC (n=5) were incubated with or without rapamycin for 48hours. P16 and P53 proteins expressions were measured by Western Blot. β-actin was used as an internal control. All results were normally distributed and shown as mean ± SEM. *P <0.05, ****P<0.0001.

In order to identify possible ways by which IHH achieved its anti-aging effect, we selected the mTOR or/and ROS pathways as keys aging-related pathways based on cues from our previous results. We then inhibited the TORC1/2 and ROS in IHH down-expressed BMSC. The results showed that the genes expressions of P53, P16, SA-β-gal, and PIK3 were compromised after inhibition of mTOR pathway and oxidative stress despite the presence of siRNA IHH (Figure 7A–7D). GDF11 results of RT-PCR presented down-regulation in gene expression after adding INK128, but not with DPI and NAC (Figure 7E). In the protein phase, we observed down-regulation of P53 and P16 associated with inhibition of mTOR and ROS pathways (Figure 8A, 8B). We also observed that BMSC morphological changes caused by IHH knockdown were improved after adding the three inhibitors (Figure 9A). Meanwhile, western blot results showed down-regulation in the phosphorylation of PI3K, Akt1, NF-κB, and STAT3 after adding of INK128, DPI, and NAC in presence of IHH siRNA (Figure 8C–8F). In addition, the cell cycle results showed that inhibition of mTOR and ROS pathways restricted the G0/G1 cell cycle arrest caused by IHH silencing (Figure 8G). Moreover, the colony forming ability of BMSC caused by IHH knockdown was improved after inhibition of mTOR and ROS in the presence of siRNA IHH (Figure 8H). Furthermore, the differentiation results revealed compromised adipogenesis with INK128, DPI and NAC treatment in IHH siRNA-transfected BMSC, presented with yellow-red fat droplets disappearance (Figure 9Ba, 9D), decreased adipocyte counts (Figure 9Bb, 9E), and decreased genes expression of adipogenic markers, PPARγ, LPL, and FABP4 (Figure 9G). On the contrary, osteogenesis improved with INK128, DPI, and NAC treatment in which the red-orange depositions were increased (Figure 9C, 9F) and genes expression of osteogenesis markers, ALP-BMA, RUNX2, and osteocalcin were up-regulated (Figure 9G). Furthermore, the specific interaction between IHH and mTORC downstream substrates was assessed. As we expected, knock down of IHH induced 4EBP1 and p70S6K1/2 phosphorylation but rIHH protein treatment down-regulate the phosphorylation process (Figure 10A). Since INK128 is the dual inhibitor for TORC1/2 downstream substrates, 4EBP1 and p70S6K1/2, we wondered to check whether IHH modulate aging-related proteins via dual or single mTOR inhibition. To perform that, siRNA IHH-transfected BMSC incubated with and without rapamycin, S6K protein antagonist to measure the expressions of P53 and P16 proteins. The results showed increased P53 and P16 expressions in cells treated with siRNA IHH but decreased after rapamycin treatment (Figure 10B). Taken together, our findings explained that IHH regulated the aging process in BMSC via dual or single modulation of the phosphorylation of TORC1/2 downstream substrates, 4EBP1 and p70S6K1/2, and down-regulation of oxidative stress.

## DISCUSSION

It is already known that Hedgehog signaling, especially IHH, plays a major role in the development of bone and cartilage, particularly at embryonic level [22, 23]. One of the major challenges in the application of MSC in the treatment of autoimmune diseases and regenerative medicine is that MSC assumes aging mode right from the onset of their multiplication in vitro [24]. In this study, we explored for the first time the anti-aging effect of IHH ligand in BMSC through suppression of mTOR and ROS pathways. Our findings showed decreased IHH expression both at the transcription and protein levels in the senescent BMSC. In literature, Hedgehog signaling has been reported to play an important role in fracture healing in young mice [25]. Also, activation of IHH is accompanied by up-regulation of trans-membranous Hedgehog receptors PTCH1/2. In the present study, we noted a declined expression of PTCH1/2 receptors, an observation which commensurate the reduced level of IHH in aged BMSC.

Consistently, PTCH1 was induced during bone formation in rat ulna [26]. The need to maintain young MSC in treatment has been underscored in a report which suggests transfusion of young MSC delays occurrence of aging in mice [27], whereas adult mice are characterized by declined progenitor frequencies [28]. These findings indicated the possible contribution of IHH signaling to the repairing and development of the skeleton, and our results demonstrated that the absence or shortage in IHH may induce early aging.

The involvement of Hedgehog signaling in protection from aging-related diseases has been reported. For example, the sonic ligand is considered a positive player in regenerative medicine, particularly in mammalian cardiac regenerative response and nerve regeneration in aged pelvic plexus [29, 30]. Affirmatively, our results showed that IHH knockdown in BMSC induces expressions of aging-related genes; P53, P16, PPARγ, mTOR, SA-β-gal and PIK3, and proteins; P53, P16, and PI3K, as well as increased SA-β-gal stained cells count. Interestingly, we found that treatment of BMSC with exogenous IHH reversed aging-related genes and SA-β-gal stained cells count. Although the role of GDF11 in aging is still an issue of conflict [31, 32], we observed that IHH silencing increased GDF11 expressions. Similar to our observation, Egerman and his coworkers found that GDF11 inhibited regeneration of skeletal muscle and accelerated aging [33]. Possibly, IHH directly or indirectly puts a check on GDF11 as a means of delaying aging in BMSC. The induction of aging-related genes and proteins following IHH knockdown suggests IHH plays an anti-aging role in BMSC and supports its potency in regeneration activities. The current study also revealed up-expression of COX-2, IDO, and IL-6 genes expressions and down-expression of HGF and TGFβ after IHH silencing in BMSC. Consistent with these findings, senescence messaging secretome (SMS) of MSC has been reported as a tool in aging regulation which triggers senescence through paracrine secretions [34, 35]. Additionally, an increase in some paracrine secretions of BMSC contributes to osteoclastogenesis, an aging-related pathway that produces osteoporosis [36, 37]. Seemingly, IHH silencing causes aging of BMSC cells population through the generation of senescence-associated secretome phenotype (SASP) which induce cell-to-cell communication by altering their paracrine secretions, as shown by the differential expression of the cytokines observed in this study.

Senescence is a biological process which occurs at the late age of eukaryotes, and it is regulated through intracellular signaling pathways. Studies show that signs of aging, which include cell cycle arrest, are induced through JAK2/STAT3 and PI3K/Akt/NF-κB signaling pathways in biliary tract malignancy [38]. In addition, intracellular ROS pathway is also involved in the aging of MSC [8, 16, 17]. In our study, we found that transfection of BMSC by siRNA IHH induced phosphorylation of PI3K, Akt1, NF-κB, and STAT3, and ROS generation, as well as induced G0/G1 phase cell cycle arrest and inhibited colonies formation. We hypothesized that IHH regulates BMSC senescence through phosphorylation modulation of the above-mentioned signaling pathways and promotes the underline mechanisms of proliferation. On the other hand, BMSC skewed differentiation is considered as one of the main causes in osteoporosis, the well-known aging-related disease due to MSC senescence [6]. In the same context, we observed induced adipogenesis associated with suppressed osteogenesis after BMSC transfection with siRNA IHH. More importantly, we discovered that exogenous IHH induces osteogenesis and suppresses adipogenesis in both senescent and non-senescent BMSC, and also compromises aging markers in SAMP8 mice, suggesting protection from aging may be exerted by IHH protein through correction of skewed differentiation.

Previous studies suggested that the mTOR and ROS signaling pathways are major factors in the eukaryotic cell aging. Nacarelli and his group reported that mitochondrial oxidative stress mediates human cells and tissues aging through activation of the mTOR/p70S6K pathway [39]. In addition, the PI3K/Akt/mTOR pathway has been reported as an activator of premature aging of preadipocytes after stimulation of ROS signaling pathway by H_2_O_2_ [40]. Moreover, mTOR and ROS signaling pathways are reported in the molecular basis of aging-related diseases especially cardiovascular diseases [41]. Furthermore, mitochondrial ROS is seen as a regulatory factor for adipogenic differentiation [42]. Recently, Robert and his co-workers reported on the implications of the mTOR signaling pathway in diseases involved in aging and differentiation [43]. In this study, we observed that aging signs in BMSC exerted by IHH silencing were compromised after inhibition of mTOR and ROS pathways by INK128 or rapamycin, and the antioxidants, DPI or NAC respectively in the presence of siRNA IHH. In addition, we discovered for the first time that the dual inhibition of 4EBP1 and p70S6K phosphorylation by the specific antagonist, INK128 in the presence of siRNA IHH normalized aging-related genes and proteins, signaling pathways, PI3K, Akt1, NF-κB, and STAT3, corrected biased differentiation, and increased BMSC cells proliferation. Also, inhibition of oxidative stress by DPI and NAC in the presence of siRNA IHH provided similar results. Taken together, IHH rejuvenates BMSC by suppression of intracellular ROS which subsequently suppresses STAT3, PI3K/Akt/NF-κB, and downregulate PI3K/Akt/mTOR pathways through interruption of 4EBP1 and p70S6K phosphorylation.

In conclusion, this study demonstrates the role of IHH as an anti-aging factor in BMSC which functions by downregulating ROS/PI3K/Akt/NF-κB/mTOR/4EBP1-p70S6K pathway. Additionally, we discovered a novel pathway which could contribute to rejuvenating BMSC cells *in vitro* to increase their regenerative and immunomodulatory potency. Moreover, this discovery could provide a therapeutic strategy in the management of aging-related diseases including osteoporosis, arthritis, cardiovascular diseases, and neurological diseases. Furthermore, our study is the first to indicate the possibility of using sapanisertib in the treatment of aging-related diseases. However, the introduction of exogenous IHH for the treatment of aging-related diseases still needs further exploration *in vivo*.

## MATERIALS AND METHODS

### Human and animal samples

Fifteen iron deficiency patients and six healthy donors were involved in this study (15 females/6males), age ranged from 25 years up to 81 years with a SD of 20.92 and mean of 54.52. The body weight ranged from 48kg to 78kg with SD of 8.39 and mean of 60.52kg. Bone marrow aspirations were collected from the Second Hospital of the Dalian Medical University, Dalian, China. SAMP8 mouse line was donated by physiology department of Dalian Medical University. The ethical committee of human and animal research of the Dalian Medical University has given the approval for this study.

### Isolation of BMSCs

Bone marrow aspirates collected in heparin anticoagulant tube were diluted in an equal volume of PBS. The mixture was dispensed slowly along the wall into a 15ml conical tube containing a double volume of Ficoll-Hypaque density gradient (Lymphoprep, TBD Science, Tianjin, China) and then centrifuged at 800g for 20 minutes continuously. The cloud-like layer of mononuclear cells was collected from the interphase (above the gray layer of Ficoll) into a new sterile tube. The collected cells were washed with sterile PBS two times by centrifugation at 1000 RPM for 5 minutes. The sediment was reconstituted aseptically with 8ml culture medium of 90% low sugar Dulbecco’s modified Eagle’s medium (DMEM) (Biological Industries, USA), 10% fetal bovine serum (FBS) (Biological Industries, USA), and 100 U/ml penicillin, and 100μg/ml streptomycin (P/S) in 75cm flask and then incubated at 37°C, 5% CO_2_ and 95% humidity overnight. Floating cells were discarded, and adherent cells washed with PBS, and medium replaced every 2-3 days. At 80-90% confluency, the BMSCs were passaged up to 3^rd^ to 5^th^ passages to be used in experiments. Isolation of mice BMSC was performed using SAMP8 mouse line. The femurs were dissected; both ends were cut and rinsed with PBS, then culture as above human BMSC.

### Generation of senescent BMSC

The senescent cells were generated from late passages 9-11 with incubation at 20% oxygen and repeated treatment by H_2_O_2_ (200μM) according to the standard protocol [40, 44]. The senescence markers are presented in Figure 1. Results of assays on senescent BMSC were compared with young cells generated from early passages (2 to 4) without H_2_O_2_ treatment.

### IHH silencing

Knockdown of IHH was performed by transfecting lipofectamine 2000 (Invitrogen, Guangzhou, China) and small interference RNAs (siRNAs) complex (Gene Pharma, Shanghai, China) sequences, shown in Table 1. Briefly, siRNA and lipofectamine were incubated separately in 150μL DMEM for 5 minutes at room temperature (RTº) and then mixed thoroughly in RNase free sterile tube. After 25 minutes of incubation at RTº, the transfection mixture was added on 60-70% confluency BMSC in 6-well plat within 1mL DMEM free FBS. The cells were used in experiments after 24 or 48 hours incubation at 37°C, 5% CO_2_, and 95% humidity. The transfection efficacy was tested by reverse transcription polymerase chain reaction (RT-PCR) and western blot. The whole process was validated using FAM negative control (Supplementary Figure 2C), GAPDH positive control, and siRNA negative control (Table 1) (Gene Pharma, Shanghai, China).

**Table 1:** Oligonucleotides of used siRNA.

**siRNA**	**Sequence (5’-3’)**
IHH-homo-296	CCCAAUUACAAUCCAGACATT UGUCUGGAUUGUAAUUGGGTT
IHH-homo-570	GCUUUGACUGGGUGUAUUATT UAAUACACCCAGUCAAAGCTT
IHH-homo-877	GCUCUUUACGGCUGACAAUTT AUUGUCAGCCGUAAAGAGCTT
Negative Control FAM	UUCUCCGAACGUGUCACGUTT ACGUGACACGUUCGGAGAATT
GAPDH Positive Control	UGACCUCAACUACAUGGUUTT AACCAUGUAGUUGAGGUCATT
Negative Control	UUCUCCGAACGUGUCACGUTT ACGUGACACGUUCGGAGAATT

### Cell culture and treatment

BMSC at 90% confluency in cell culture flasks were digested with trypsin EDTA 0.25%, passaged, and propagated with 10% FBS in DMEM and 100 U/ml/100μg/ml P/S, and incubated at 37ºC with 5% CO_2_ and humidity. BMSC typical morphological characters were captured under a phase contrast microscope within 3 to 5 passages. Identification of BMSC cells were performed using primary antibodies conjugated with fluorescein isothiocyanate (FITC), phycoerythrin (PE), or phycoerythrin-cyanin 5/7 (PC5/7) against CD14, CD34, CD45, CD73, CD90, CD105, and CD140a by Accuri C6 flow cytometer (BD Biosciences, San Jose, CA, USA) (Supplementary Figure 1). BMSC were treated with recombinant IHH (rIHH) 300ng/ml (Prospec, USA). While BMSC were transfected by IHH siRNA, they were treated at the same time with 200nM sapanisertib (INK128) mTOR inhibitor (Cayman Chemical, USA), 300 nM rapamycin (Solarbio, Beijing, China), 5μM diphenyleneiodonium chloride (DPI) ROS inhibitor (Sigma-Aldrich, USA), or 5mM N-acetyl-L-cysteine (NAC) ROS inhibitor (Sigma-Aldrich, USA) in serum-free DMEM for 24hours in all experiments. INK128 and rapamycin efficiency were evaluated as shown in Figure 10A.

### SA β-gal stain

60% confluency BMSC in 12-wells plate were washed with PBS and stained using SA β-gal staining kit (Solarbio, Beijing, China) according to the factory’s instructions. Briefly, the cells were incubated at RTº with a 0.5ml fixative solution (A) for 15 minutes and then washed three times by PBS, 3 minutes for each with gentle shaking. Staining working reagent (C) was prepared by mixing 5μl dye solution A, 5μl dye solution B, 465μl dye solution C, and 25 μl X-gal solution (B) for each well. The cells were incubated with 0.5ml staining working reagent for 6 hours at 37°C without CO_2_ and then observed under invert microscope (200X total magnification) to measure the green-blue spots.

### mRNA extraction and RT-PCR

Extraction of mRNA from BMSC was performed using RNAiso (Takara Bio, Japan) as explained in a previous article [45]. PrimeScript^™^ 1^st^ strand cDNA Synthesis Kit (Takara Bio, Japan) was used to produce cDNA using 1μg of extracted mRNA. Primers shown in Table 2 were used in PCR amplification and glyceraldehyde 3-phosphate dehydrogenase (GAPDH) was used as a housekeeping gene. PCR MasterMix 2X Power Taq (Bio Teke Corporation, China), cDNA, RNase Free ddH_2_O, and primers (Takara Bio, Japan) were mixed well in micro-tubes according to the manufacturer’s protocol and then loaded in the thermal cycler (Bio-Rad, USA), each cycle consisted of 30s for denaturation at 95 °C, 30s of annealing at 56.0, 56.5, 57, 57.5, or 58.0 °C, and 30s for extension at 72 °C, for a total of 35 or 40 cycles. The PCR outputs of all genes were dispensed in 2% agarose gels wells for electrophoresis in tris acetic acid EDTA (TAE) buffer. PCR bands were quantified by Image Lab detection system (Bio-Rad, USA) and Image J (ImageJ2x, Rawak Software Inc., Germany).

**Table 2:** Primers sequences used in gene amplification.

**Gene**	**Forward Primer**	**Reverse Primer**
GAPDH (272 bp)	TGACCACAGTCCATGCCATCAC	CGCCTGCTTCACCACCTTCTT
IHH(247 bp)	GAACTCGCTGGCTATCTCGG	CTCGGACTTGACGGAGCAAT
PTCH1(298 bp)	TGTCGCACAGAACTCCACTC	ACCAAGAGCGAGAAATGGCA
PTCH2(431 bp)	TTACCGCAACTGGCTACAGG	CGATGGCCTCCACAAAGTCT
P53 (463 bp)	GCTTGCAATAGGTGTGCGTC	AAACTACCAACCCACCGACC
CDKN2A (221 bp)	CCACCCCGCTTTCGTAGTT	CCACATGAATGTGCGCTTAGG
PPARγ (513bp)	TTTGGGATCAGCTCCGTGG	CATCCGCCCAAACCTGATG
mTOR (541)	CTTAGAGGACAGCGGGGAAG	TCAGCGGTAAAAGTGTCCCC
PIK3 (207 bp)	ACTCACCTTCTGCTCCGTTG	TCATACTCGCGGCTCTTGTC
GDF11 (546 bp)	GACCAAGCCGTGTGCAATAC	AAGGGATAAACGGGGCACAG
COX-2 (146 bp)	TGACCACAGGCAGATGAA	CCACAGCATCGATGTCACCATAG
IDO1 (159 bp)	GAATGGCACACGCTATGGAA	CAGACTCTATGAGATCAGGCAGATG
IL-6 (234bp)	CCTTCGGTCCAGTTGCCTTCTC	CCAGTGCCTCTTTGCTGCTTTC
HGF (147 bp)	GTCAGCCCTGGAGTTCCATGATA	AGCGTACCTCTGGATTGCTTGTG
TGFβ (130 bp)	AGCGACTCGCCAGAGTGGTTA	GCAGTGTGTTATCCCTGCTGTCA
SA-β- gal (268 bp)	CGACTATGATGCCCCACTGA	TGTCCGGTACAGCACAAACC
FABP4 (149 bp)	ATGGGGGTGTCCTGGTACAT	ACGTCCCTTGGCTTATGCTC
LPL (473 bp)	AGTAGCAGAGTCCGTGGCTA	GGGACCCTCTGGTGAATGTG
ALP-BMA (339 bp)	CGGAAGACACTCTGACCGTG	GGACGTAGTTCTGCTCGTGG
RUNX2 (434 bp)	CAGTGGCCCAGTGGTATCTG	GAGGGCACTGGTCCATACAC
Osteocalcin (277 bp)	ACCATGAGAGCCCTCACACTC	CCTCCTGAAAGCCGATGTGG

### Western blot

Proteins were extracted from more than 10^6^ harvested BMSCs according to the kit protocol (KeyGen Biotech, China). [46] BMSCs were digested in a lysis buffer cocktail and centrifuged at 12000 g for 15 minutes at 4 °C to get the supernatant as the total protein. 10% sodium dodecyl sulfate-polyacrylamide gel (SDS-PAGE) was used for electrophoresis of protein (20 μg) with loading buffer and then blotted to a polyvinylidene fluoride (PVDF) membrane (Millipore Co., USA). Membranes were blocked with 5% skimmed milk in Tris-buffered saline tween 20, TBST, and then incubated overnight at 4°C with primary antibodies; anti-IHH, anti-P53, anti-P16, anti-PI3K, anti-p-PI3K, anti-Akt 1, anti-p-Akt 1, anti-NF-κB, anti-p-NF-κB-p65, anti-STAT3, anti-p-STAT3, anti-4EBP1, anti-p- 4EBP1, anti-p70S6K, anti-p-p70S6K and anti-β-actin (1:1000), all from Abcam, USA. The membranes were incubated for 1hour at RT with secondary antibody conjugated with horseradish peroxidase. Finally, TBST-washed membranes were treated with enhanced chemiluminescent (ECL) for detection (Bio-Rad, USA). Image Lab detection system (Bio-Rad, USA) was used for protein bands imaging and analysis.

### BMSC *in vitro* bi-lineage differentiation assay

BMSCs in 24-well plate of 80% confluency were incubated with 10% FBS in adipogenic or osteogenic differentiation medium containing 100 U/ml/100μg/ml P/S for 21 days at 37°C with 5% CO_2_ and humidity. The medium was replaced every 3 days during the above-mentioned period. For adipogenesis, the medium was designed as a following, 0.5 mM 1-methyl-3-isobutylxanthine, 1μM dexamethasone, 10μg/ml insulin, and 0.2 μM indomethacin in MEM-ALPHA medium (Biological Industries, USA). Adipogenesis was measured by the intensity of lipid vacuoles or droplets accumulations which appear yellow-red after staining with Oil-Red-O lipophilic dye (Coolaber, China), and by visualization of adipocytes using inverted light microscopy prior staining. For osteogenesis, the medium was prepared as a following, 0.1μM dexamethasone, 0.05mM ascorbic acid, and 10mM Tumor protein p53 (TP53), cyclin-dependent kinase inhibitor 2A (CDKN2A), peroxisome proliferator activated receptor gamma (PPARγ), growth differentiation factor 11 (GDF11), cyclooxygenase 2 (COX-2), indoleamine 2,3-dioxygenase 1 (IDO1), interleukin 6 (IL-6), hepatocyte growth factor (HGF), transforming growth factor beta 1 (TGFβ), senescence associated β-galactosidase (SA-β-gal), fatty acid binding protein 4 (FABP4), lipoprotein lipase (LPL), alkaline phosphatase, biomineralization associated (ALP-BMA), runt related transcription factor X2 (RUNX2).

glycerophosphate in MEM-ALPHA medium (Biological Industries, USA). Osteogenesis and mineralization were assessed by the intensity of calcium deposition which stains red-orange by Alizarine-Red-S stain (Coolaber, China).

### ROS measurement

2^/^,7^/^-Dichlorofluorescein diacetate (DCFHDA; Sigma-Aldrich, USA) was used to quantify ROS generation levels. 5μM DCFHDA and 70% confluency BMSC were incubated in DMEM at 37°C with 5% CO_2_ and humidity for 30 min. Cells were harvested after being washed two times with PBS and quantified by Accuri C6 flow cytometer (BD Biosciences, San Jose, CA, USA) at 488 nm and 538 nm wavelength. Fluorescence microscope (Olympus1X71, Japan) was also used to view and capture images of stained cells.

### Cell cycle assay

Harvested washed one million BMSC suspended in 1mL PBS were fixed by adding them slowly drop by drop in 9mL 75% ethanol while vortexing. Cells in 75% ethanol were incubated overnight at 4°C and then the pallet was reconstituted after centrifugation in 200μL of a mixture containing 20 μg/mL propidium iodide (PI; BD Biosciences, USA), and 10 μg/mL RNase-A (Coolaber, China) in 0.2% Triton-x water. The stained cells were incubated 15 minutes at RT° and then analyzed using flow cytometry Accuri C6 (BD Biosciences, San Jose, CA, USA).

### Fibroblast colony forming unit assay (F-CFU)

3000 BMSC were seeded in complete medium 10% FBS in MEM-ALPHA and 100 U/ml/100μg/ml P/S for 12 days at 37°C 5% CO_2_ and humidity. The cells were washed with PBS, fixed with 1% paraformaldehyde, and stained with 0.2% crystal violet. The average colony count was estimated from images taken from at least five different fields per well.

### Statistical analysis

Analysis of data was done by GraphPad Prism Version 6.07 (San Diego, USA). Comparison of variables was achieved with either one-way analysis of variance (ANOVA) or paired-sample t-test. All experiments were performed at least in triplicates, and data presented as mean ± SD. The differences between groups were considered statistically significant once *p-value* was < 0.05.

## Supplementary Material

Supplementary Figures
