# Supplementary material for: Indian Hedgehog regulates senescence in bone marrow-derived mesenchymal stem cell through modulation of ROS/mTOR/4EBP1, p70S6K1/2 pathway
Source: Aging (Albany NY). 2020 Apr 1;12(7):5693–715. doi: 10.18632/aging.102958 (PMC7185126; doi:10.18632/aging.102958)
Supplement: Supplementary Figures [file aging-12-102958-s001..pdf]

SUPPLEMENTARY FIGURES

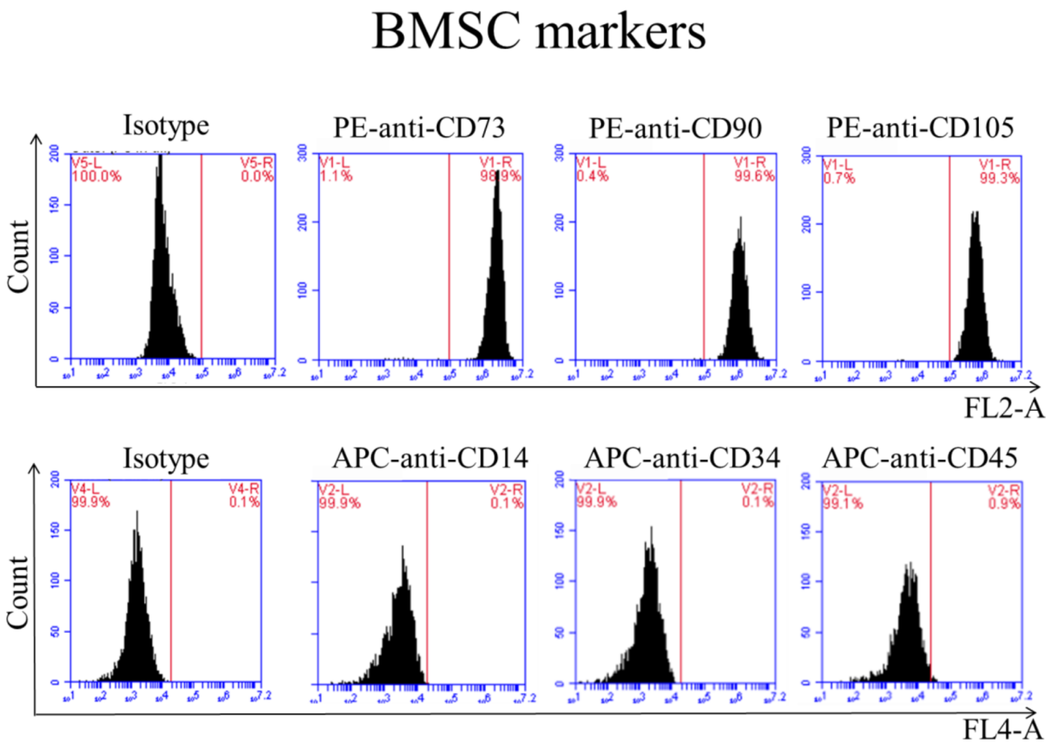

Supplementary Figure 1. Surface markers expressions of BMSCs. BMSC (n=3) were incubated with antibodies against CD14, CD34, CD45, CD73, CD90, and CD105 for 30 minutes then analyzed by Accuri C6 flow cytometer. The figure showed that CD73, CD90, and CD105 markers were positive, whereas CD14, CD34, and CD45 markers were negative.

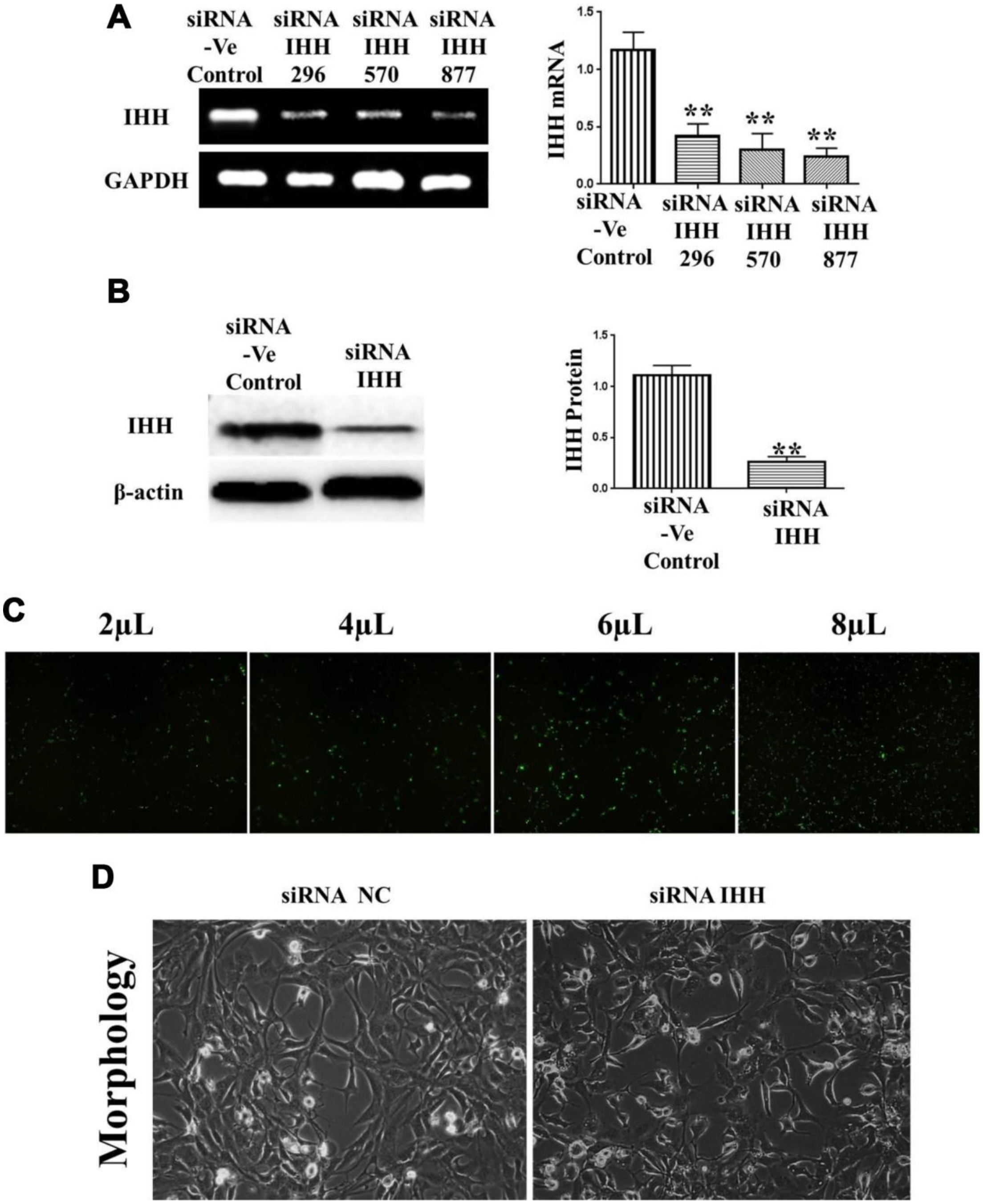

Supplementary Figure 2. Silencing of IHH in BMSC. (**A**) BMSC (n = 6) were transfected with siRNA negative control or siRNA IHH for 24hours. IHH gene expression was measured by RT-PCR. GAPDH was used as a housekeeping gene. (**B**) BMSC (n = 6) were transfected with siRNA negative control or siRNA IHH for 48hours. IHH protein expression was measured by Western Blot. β-actin was used as an internal control. (**C**) Different concentrations of FAM siRNA negative control in BMSC visualized under invert microscope (**D**) Morphology of BMSC with and without IHH siRNA.

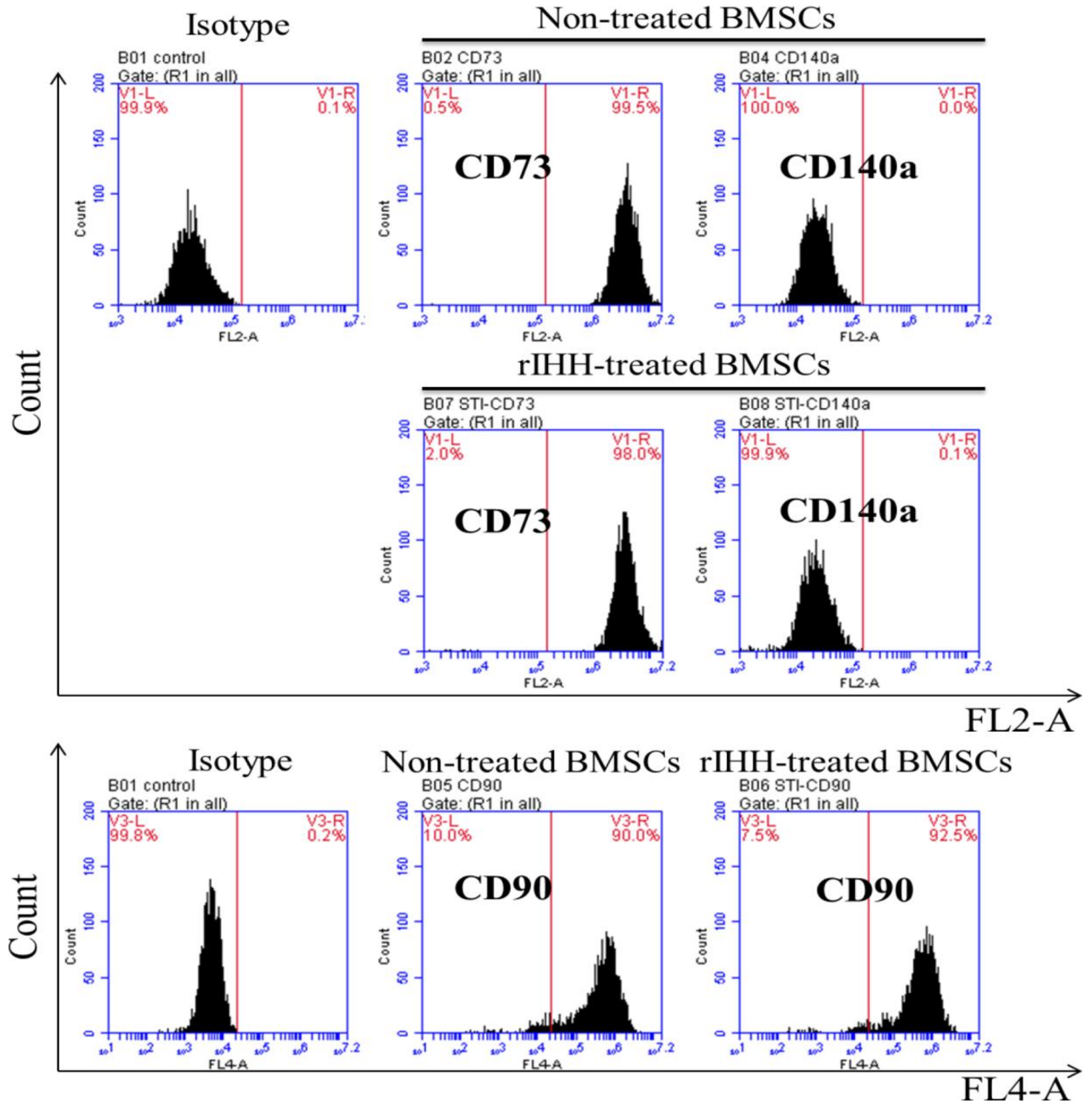

Supplementary Figure 3. The effect of rIHH on surface markers expressions of BMSCs. BMSC (n=3) were incubated with rIHH overnight then with antibodies against CD73, CD90, and CD140a for 30 minutes then analyzed by Accuri C6 flow cytometer. The figure showed that CD73, CD90, and CD140a were not affected by rIHH treatment.
